# Diversity and structural analysis of rhizosphere soil microbial communities in wild and cultivated Rhizoma Atractylodis Macrocephalae and their effects on the accumulation of active components

**DOI:** 10.7717/peerj.14841

**Published:** 2023-02-16

**Authors:** Pingping Song, Junling Liu, Peng Huang, Zhili Han, Dianlei Wang, Nianxia Sun

**Affiliations:** 1 School of Pharmacy, Anhui University of Chinese Medicine, Hefei, China; 2 Key Laboratory of Quality Research and Evaluation of Traditional Chinese Medicine, State Medical Products Administration, Hefei, China; 3 Anhui Province Key Laboratory of Research and Development of Chinese Medicine, Hefei, China

**Keywords:** Rhizoma Atractylodis Macrocephalae, Wild and cultivated, Rhizosphere microbial community, Rhizosphere fungi, Bacterial community, Root chemical composition accumulation

## Abstract

Rhizosphere microorganisms are the main factors affecting the formation of high quality medicinal materials and promoting the accumulation of secondary metabolites. However, the composition, diversity, and function of rhizosphere microbial communities in endangered wild and cultivated *Rhizoma Atractylodis Macrocephalae* (RAM) and their relationships with active component accumulation have remained unclear. In this study, high-throughput sequencing and correlation analysis were used to study the rhizosphere microbial community diversity (bacteria and fungi) of three RAM species and its correlation with the accumulation of polysaccharides, atractylone, and lactones (I, II, and III). A total of 24 phyla, 46 classes, and 110 genera were detected. The dominant taxa were *Proteobacteria*, *Ascomycota*, and *Basidiomycota*. The microbial communities in both wild and artificially cultivated soil samples were extremely species-rich, but there were some differences in their structure and the relative abundances of microorganism taxa. Meanwhile, the contents of effective components in wild RAM were significantly higher than those in cultivated RAM. Correlation analysis showed that 16 bacterial and 10 fungal genera were positively or negatively correlated with active ingredient accumulation. These results showed that rhizosphere microorganisms could play an important role in component accumulation and might lay a foundation for future research on endangered materials.

## Introduction

The plant rhizosphere, as the micro-environment between the root system and soil interface, plays an important role in soil-root-microbe interactions (Butler et al., 2003). In such particular area, a large number of plant-related microbial communities are inhabited (Edwards et al., 2015; Turner, James & Poole, 2013), including bacteria, fungi, archaea, protozoa, and other functionally unknown microorganisms, and are considered as the second genome of plants (Badri et al., 2009). Rhizosphere microorganisms are an important part of the plant rhizosphere micro-ecosystem, and their population structure and activity changes are important indicators used for measuring soil quality, maintaining soil fertility, plant stress resistance, and disease inhibition (Zhou, Tang & Guo, 2018; Zhou et al., 2021). Furthermore, it has been shown that rhizosphere microorganisms can affect the synthesis of good quality medicinal materials (Berendsen, Pieterse & Bakker, 2012; Qi et al., 2012), which can promote the accumulation of officinal effective ingredients. It had been reported that inoculation of Arbuscular mycorrhizal fungi could promote plant growth and the production of bioactive components in medicinal plants (Zeng et al., 2013). Pseudomonas, Bacillus and Mycorrhizal fungi which were isolated from the rhizosphere, could promote the production of secondary metabolites (Lugtenberg & Kamilova, 2009). In the previous study, the correlation between angelica rhizosphere microorganisms and the accumulation of phthalic acid compounds was analyzed, and it had been revealed that Bacillus could promote the accumulation of butene phthalides (Feng et al., 2021). It was of great significance to study the rhizosphere microorganisms of medicinal plants and their impacts on the accumulation of active ingredients in these medicinal plants.

Rhizoma Atractylodis Macrocephalae (RAM), frequently referred to as “Baizhu” in Chinese, is the dried root of RAM, a Compositae plant. RAM has a history, which is more than two thousand years in China. It has traditional effects such as treating spleen dysfunction, loss of appetite, abdominal distension and diarrhea, phlegm and vertigo, palpitation, edema, and fetal movement (Zhu et al., 2018). Phytochemical research has revealed that RAM mainly contains three categories of compounds, volatile oil, lactones, and polysaccharides (Yang et al., 2002; Duan et al., 2008). Pharmacological studies have indicated that RAM possessed antitumor activities, neuroprotective effects, anti-hepatotoxicity, immune and anti-inflammatory activity, *etc*. (Ruqiao et al., 2020). RAM has been introduced and cultivated in most parts of the country, with Yuqian city in Zhejiang Province and the southern Anhui mountainous areas as the traditional top-geoherb region due to the excellent quality, superior clinical effects, and good market reputation. The resources of RAM in Anhui Province have been developed and utilized since the Song Dynasty. At present, Bozhou city is the main base of artificial planting areas, and its RAM is usually known as “Bozhu”. Wild RAM is mainly distributed in Qimen city and Shexian city, being often referred to as “Qizhu” and “Shezhu”, respectively. Although Bozhu, Qizhu, and Shezhu belonged to the same genus, there were significant differences in smell, flowering dynamics, leaf dynamics, and so on. Interestingly, their ingredients and efficacies were also inconsistent. The short growth period, the loose texture of medicinal materials and the low content of volatile oil and other active components of Bozhu might lead to the poor quality of RAM cultivated in this area, which restricted the development of RAM. Correspondingly, wild Shezhu and Qizhu samples had more excellent medicinal value and a wider application range (Cui et al., 2020). However, the wild resources of RAM were very endangered. The current source of RAM for clinical use is mostly artificial cultivation. Therefore, promoting the accumulation of effective components and improving the quality of RAM medicinal materials might be the key to being solved urgently.

Currently, research on the effective components of RAM mainly focused on the separation and identification of chemical components (Zhao et al., 2017), quality control (Zheng et al., 2021), and pharmacological activities (Yang et al., 2021). In terms of the microecological environment, most of the research focused on the effects of environmental factors (climate, soil characteristics, and water quality) and associated endophytic bacteria on the active ingredients of RAM (Zhu et al., 2020; Zhang et al., 2015). However, the composition, diversity, and function of rhizosphere microbial communities of endangered authentic and artificial cultivation of RAM, as well as their effects on the accumulation of polysaccharides, atractylone, and lactone I, II, and III, were still unclear.

In this study, the main purpose was to reveal the correlation between rhizosphere microbial communities and the effective components accumulation of RAM from endangered authentic sources (Qizhu and Shezhu) and artificially cultivated sources (Bozhu) in Anhui Province. Illumina MiSeq and 16S rRNA high-throughput sequencing technology were used to determine and compare the composition and structure of the rhizosphere microbial community of RAM from different habitats, to clarify the characteristics of the rhizosphere microbial community of RAM based on classification operation unit (OTU). Meanwhile, the phenol-sulfuric acid method and high-performance liquid chromatography (HPLC) were used to determine the contents of polysaccharides, atractylone, and lactone I, II, and III compounds in RAM. Through correlation analysis, the assembly rules between rhizosphere microorganisms and the accumulation of polysaccharides, atractylone, and lactones in RAM were revealed. It could provide a theoretical basis for rhizosphere microorganisms to promote the accumulation of active ingredients in medicinal materials.

## Materials and Methods

### Soil sample collection

In this study, three different producing areas of RAM were selected as sampling points in Qimen and Shexian County of Huangshan City and Qiaocheng District of Bozhou City in Anhui Province. The sampling areas in Qimen and Shexian counties belong to the north subtropical monsoon climate (N29°35′, E117°57′). With an altitude of 1,000 m, the terrain is relatively high, and the climate is mild. It was located in a cloudy and foggy hillside woodland shrub forest, mainly growing in humus soil, showing a small individual population, only two or three plants grow together, and often mixed with a variety of plants. The samples collected are three or five year old samples. Qiaocheng District of Bozhou City has a temperate monsoon climate (N33°88′, E115°77′), with an altitude of 25 m and flat terrain. RAM samples are artificially cultivated products, planted in a large range, mostly annual or biennial medicinal materials.

Each sample was collected using a five-point sampling method, with each type of soil sample coming from at least three healthy, disease-free RAMs. All the samples are carefully removed from the surrounding soil sampling sites. During sampling, soil humus was removed first, and roots of healthy plants were taken by digging 0–20 cm vertically along the root of RAM. The non-rhizosphere soil attached to the root was gently shaken off by hand. Then the residual soil about 2 mm away from the roots was brushed away using a sterile spatula. The soil samples were mixed in equal amounts, placed in a sterile plastic bag, bag marked sampling site, quickly stored in an ice box, and brought back to the laboratory sieve (80 mesh), −80 °C stored for later use (Dong et al., 2021). RAM samples from different origins were marked as Qizhu (A1–A7), Shezhu (B1–B4), and Bozhu (C1–C5).

### DNA extraction, PCR amplification

According to the manufacturer’s instructions, genomic DNA from rhizosphere soil of RAM from three producing areas was extracted using PowerSoil DNA Isolation Kit (MoBio Laboratories, Carlsbad, CA, USA ) (Li et al., 2022b). The bacterial 16S rRNA gene sequencing and the fungal ITS gene sequencing were proceed as described previously (Yuan et al., 2020).

### High throughput sequencing

Paired-end sequencing was performed using Illumina Miseq PE300 high-throughput sequencing platform in Beijing Ovidson Gene Technology Co., Ltd. Original sequence uploaded to NCBI database (Li et al., 2022a).

### Sequence processing

The off-machine data was split by the QIIME1 (v1.8.0) software according to the Barcode sequence, and the Pear (v0.9.6) software was used to filter and splice the data. After splicing, Vsearch (v2.7.1) software was used to remove sequences less than 230 bp in length, and chimera sequences were removed by the uchime method alignment according to the Gold Database (Wang et al., 2018). Finally, the Vsearch (v2.7.1) software uparse algorithm was used to perform OTU clustering (Operational Taxonomic Units) on the high-quality sequences, and the similarity threshold was 97% (Edgar, 2013). Compared with the Silva128 database using the RDP Classifier algorithm, a 70% confidence threshold was set to obtain the species classification information corresponding to each OTU (Cole et al., 2009). We then used QIIME1 (v1.8.0) software for alpha diversity index analysis (including Shannon, Simpson, and Chao1 indexes). Based on species annotation and relative abundance results, R (v3.6.0) software was used to perform a histogram analysis of species composition. QIIME1 (v1.8.0) was used to calculate the beta diversity distance matrix, and R (v3.6.0) software was used to obtain a clustering heat map and for PCoA analysis based on weighted Unifrace distance (Jiang et al., 2013). Intergroup heterogeneity analysis of metastats was performed using Mothur (v.1.34.4) software and LEfSe analysis was performed using Python (v2.7) software (Jami et al., 2013).

### Determination of polysaccharides, atractylone, and lactones components in the samples

As described in the materials and methods, three producing areas of RAM picking back to the laboratory. After removing the rhizosphere soil, the root of RAM root herbs was washed and sliced after low-temperature drying. After drying, the RAM medicinal materials were placed in a pulverizer to crush them and passed through a No. 3 sieve. The contents of lactone I, II, III and atractylone in different habitats were determined by HPLC. The content of polysaccharides was determined by the phenol-sulfuric acid method. We obtained 1 g of medicinal powder and placed it in a 10 mL volumetric flask, followed by the addition of methanol (chromatographic grade) to a constant volume to of 10 mL. Samples were cleaned with an ultrasonic machine (frequency 40 Hz, power 100 W, ultrasonic time 60 min). After cooling, the supernatant was centrifuged at 10,000 rpm for 10 min with a sterile syringe and then filtered through a 0.22 μM filter into a 2.0 mL sample bottle. We dissolve 1.3, 2.3, 2.28, and 3.6 mg of lactone I, II, III, and atractylone standard in 1 mL of methanol to achieve concentrations of 1.3, 2.3, and 2.28, and 3.6 mg/g, respectively. Chromatographic conditions: Dikma kromasil C18 (250 × 4.6 mm, 5 μm) column, acetonitrile (A)-water (B) as mobile phase, gradient elution: 0–16 min, 60–76% A, 16–18 min, 76–100% A, 18–30 min, 100% A. Volume flow rate was 1.0 mL/min and column temperature was 30 °C. The detection wavelengths were 220 nm (lactone I, III), 276 nm (lactone II), and the injection volume was 10 μL.

### Statistical analysis

Statistical analysis was performed using SPSS 22.0 (SPSS, Inc., Chicago, IL, USA). One-way ANOVA was used to analyze the significant differences between the contents of polysaccharides, atractylone, and lactones from different origins. Means were analyzed using Tukey’s test. The Canoco 5 software was used to analyze the relationship between polysaccharides, atractylone, and lactones and rhizosphere soil microbial communities. *P* < 0.05 was considered to denote significant differences. All data were expressed as mean ± standard deviation.

## Results

### Comprehensive analysis of sequencing data of rhizosphere microbial diversity of RAM from three habitats

**Figure 1:**
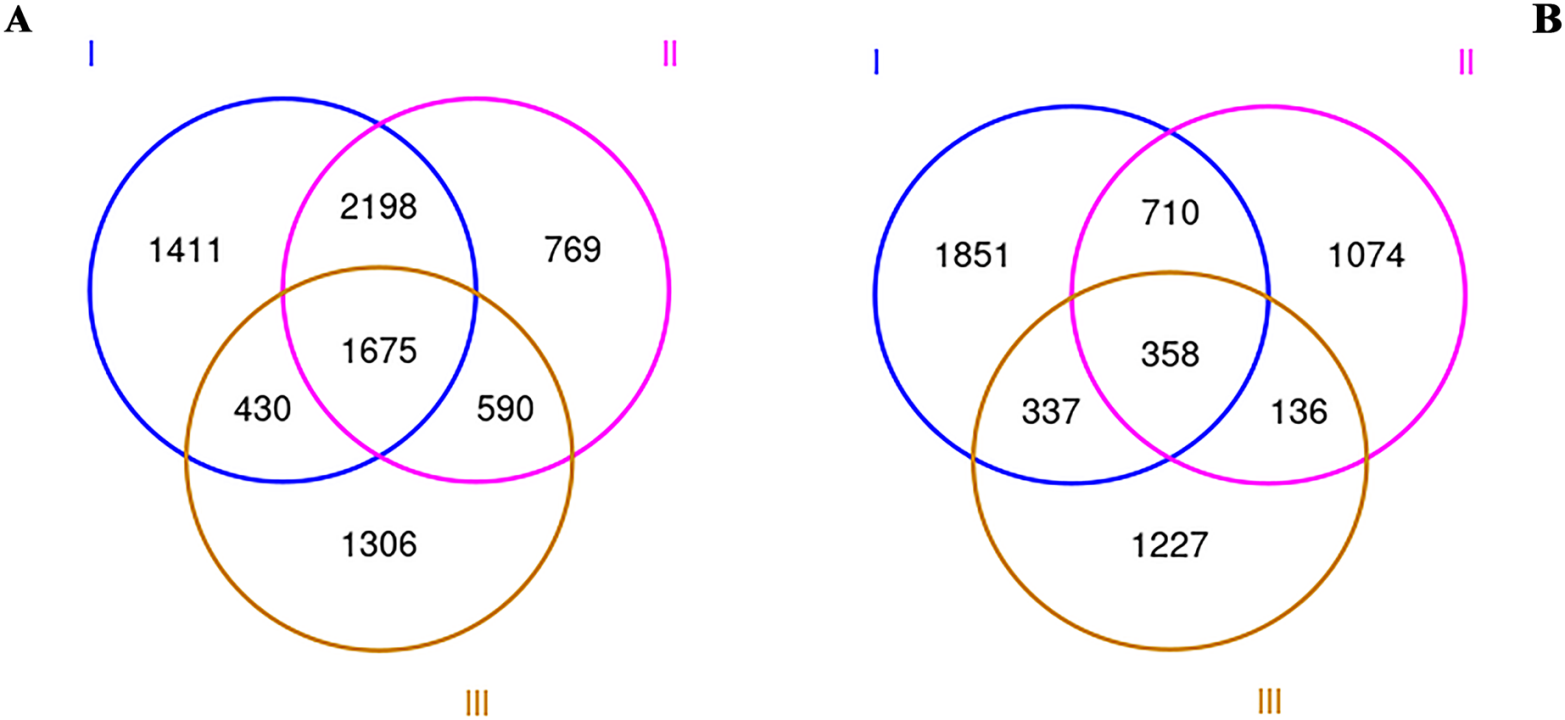
Venn diagrams created using OTUs of wild and cultivated RAM soils. (A) Bacteria; (B) fungi. I, Wild Qizhu rhizosphere soil sample; II, wild Shezhu rhizosphere soil sample; III, artificial cultivation of Bozhu rhizosphere soil sample.

To explore the rhizosphere microbial community of RAM, soil samples from three origins were collected and sequenced by Illumina MiSeq. After all sequence denoising and quality filtering, the quality-filtered sequences were clustered into 14,072 different OTUs at the 97% similarity level. All our samples are based on the statistics after flattening. The OUT-Venn map directly reflected the common and unique OTUs in all three samples. As shown in Fig. 1, a total of 8,379 and 5,693 OTUs for bacteria (a) and fungi (b) were obtained respectively. The total numbers of bacterial and fungi OTUs in the wild Qizhu sample were 5,714 and 3,256 respectively, which were higher than those of the cultivated Bozhu sample and wild shezhu sample. The total numbers of bacterial and fungi OTUs in Shezhu were the lowest among the three types. Among them, only 1,675 bacterial OTUs and 358 fungal OTUs were common in three soils.

### Alpha diversity of bacterial and fungal communities in the rhizosphere soil of RAM from the three origins

**Figure 2:**
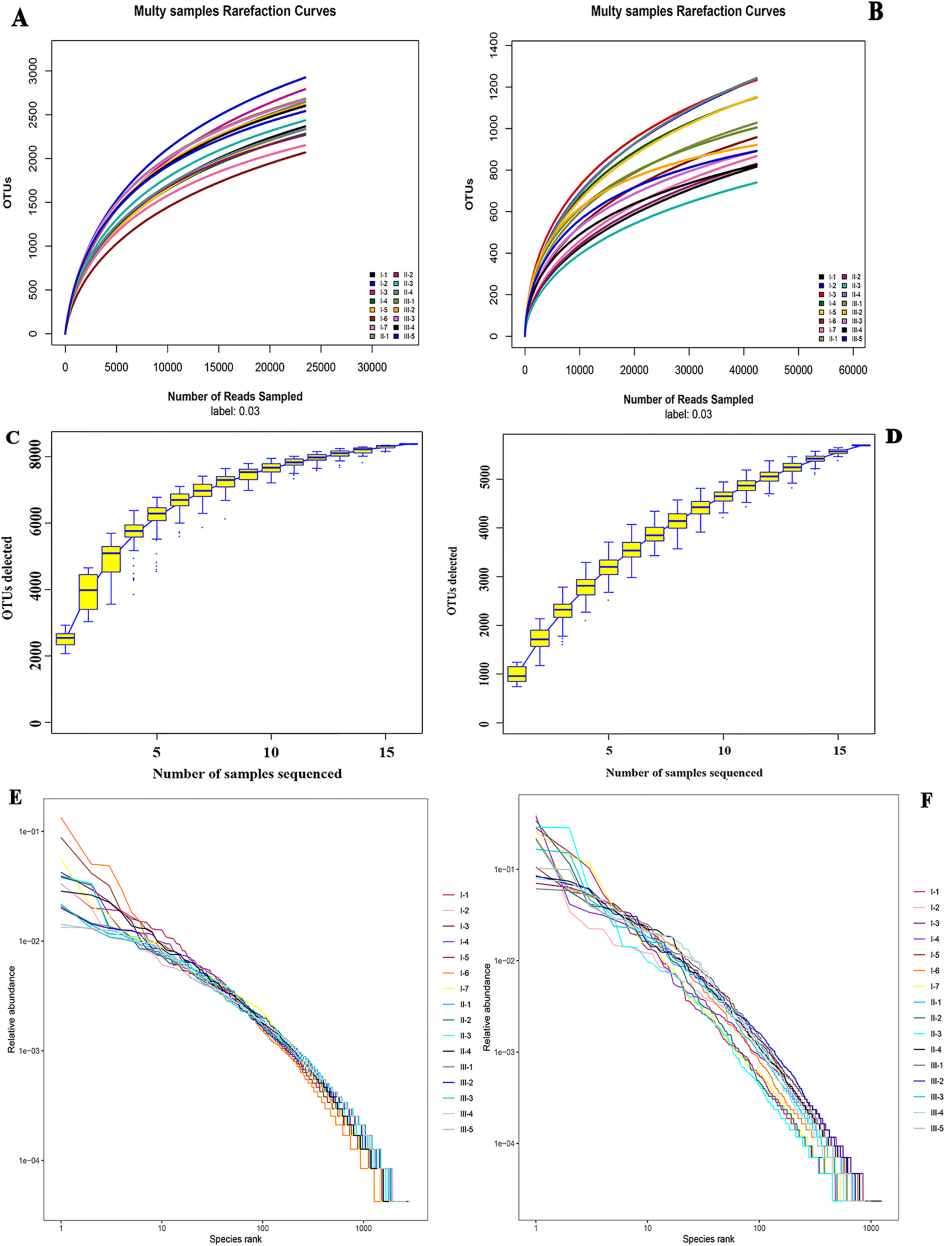
α diversity analysis. (A and B) Rarefaction curves showing the depth of sequencing and the possibility of diversity. (C and D) Specaccum species cumulative curve showing the rate of increase of new species with the increase in sample size. The upper and lower lines reflected the confidence interval for the curve. (E and F) Rank abundance curve showing the relative species abundance. The length of the polyline on the horizontal axis reflected the number of OTUs in the sample and represented the richness of the community. The longer the polyline was, the more the number of OTUs was in the sample. The flatness of the polyline reflected the evenness of the community composition. The more the polyline, the higher the homogeneity of the community composition and the steeper the polyline, representing the greater difference in abundance among the OTUs in the community and the lower evenness. I1–I7, Wild Qizhu rhizosphere soil sample; II1–II7, wild Shezhu rhizosphere sample; III1–III7, artificial cultivation of Bozhu rhizosphere soil sample. (A, C, E) Bacteria; (B, D, F) fungi.

Alpha diversity analysis was performed on species richness and uniformity within a single microbial ecosystem. Rarefaction curves showed that the higher the sequencing depth, the greater the likelihood of higher diversity observed. The OTU number curves of all samples tended to be flat, suggesting that the sequencing depth of all rhizosphere soil samples was reasonable. And it could reflect reliably the bacterial and fungal community structures and compositions in wild authentic Qizhu, Shezhu, and artificially cultivated Bozhu rhizosphere soil samples (Figs. 2A and 2B). In addition, the sample size was sufficient to reflect the richness of microbial communities (Figs. 2C and 2D). To check the relative species abundance, R software was used to draw the grade abundance curve. The abundances of Shezhu and Qizhu were high and uniform, while the abundances of Bozhu were significantly different between OTUs and low uniformity (Figs. 2E and 2F). Furthermore, the coverage indexes of all samples in Table 1 were greater than 96%, which also showed that the sequencing depth of all samples in this study was reasonable and further results were reliable.

**Table 1:** Alpha diversity indexs of soil bacteria, fungus from different habitats.

Simple ID	Chao 1		Goods-coverage		Observed-species		Shannon		Simpson	
	Bacteria	Fungi	Bacteria	Fungi	Bacteria	Fungi	Bacteria	Fungi	Bacteria	Fungi
I-1	3136.74	1241.64	0.97	0.99	2366.00	817.00	9.13	4.68	0.99	0.88
I-2	3924.29	1821.55	0.96	0.99	2924.80	1237.80	9.87	5.50	1.00	0.83
I-3	3821.18	1628.77	0.96	0.99	2791.80	1234.00	9.48	6.93	0.99	0.98
I-4	3088.10	1560.27	0.97	0.99	2282.70	1151.00	9.13	5.41	0.99	0.85
I-5	3400.19	1577.04	0.96	0.99	2340.70	1153.00	8.74	6.68	0.99	0.97
I-6	2984.50	1363.89	0.97	0.99	2069.00	958.00	8.10	5.77	0.97	0.94
I-7	2798.47	1270.58	0.97	0.99	2151.70	866.90	9.08	4.84	0.99	0.89
II-1	3583.22	1460.37	0.96	0.99	2646.70	1027.80	9.78	6.24	1.00	0.94
II-2	2913.76	1311.07	0.97	0.99	2269.60	829.90	9.33	4.79	1.00	0.86
II-3	3172.40	1107.59	0.97	0.99	2434.50	740.00	9.38	4.20	0.99	0.83
II-4	3081.41	1790.18	0.97	0.99	2334.60	1243.00	9.26	6.67	1.00	0.87
III-1	3427.55	1273.55	0.97	0.99	2681.80	1005.00	9.84	6.91	1.00	0.88
III-2	3383.58	1101.58	0.97	1.00	2615.60	921.00	9.72	6.85	1.00	0.98
III-3	3400.69	1227.03	0.97	0.99	2670.00	892.00	9.85	5.92	1.00	0.94
III-4	3304.09	1107.12	0.97	0.99	2600.50	824.00	9.80	6.40	1.00	0.97
III-5	3247.55	1084.86	0.97	0.99	2540.80	892.00	9.81	6.48	1.00	0.97

Note:

Each processing sample consists of three parallel samples. I-1-I-7, II-1-II-4, and III-1-III-5 represent rhizosphere soil of Qizhu, Shezhu, and Bozhu, respectively.

There were many indexes reflecting the alpha diversity of microbial communities. Among them, chao1 and observed-species indexes reflected the richness of communities, and Shannon and Simpson indexes reflected the diversity and uniformity of community. The greater the Shannon index, the higher the community diversity, while the greater the Simpson index, the lower the community diversity. The microbial community richness and diversity indices are listed in Table 1, the Turkey test was used to calculate *P* values after a pairwise comparison of the values corresponding to each index, and there were no significant differences in the above indicators (*P* > 0.05).

### Composition and structure of rhizosphere soil microbial community

**Figure 3:**
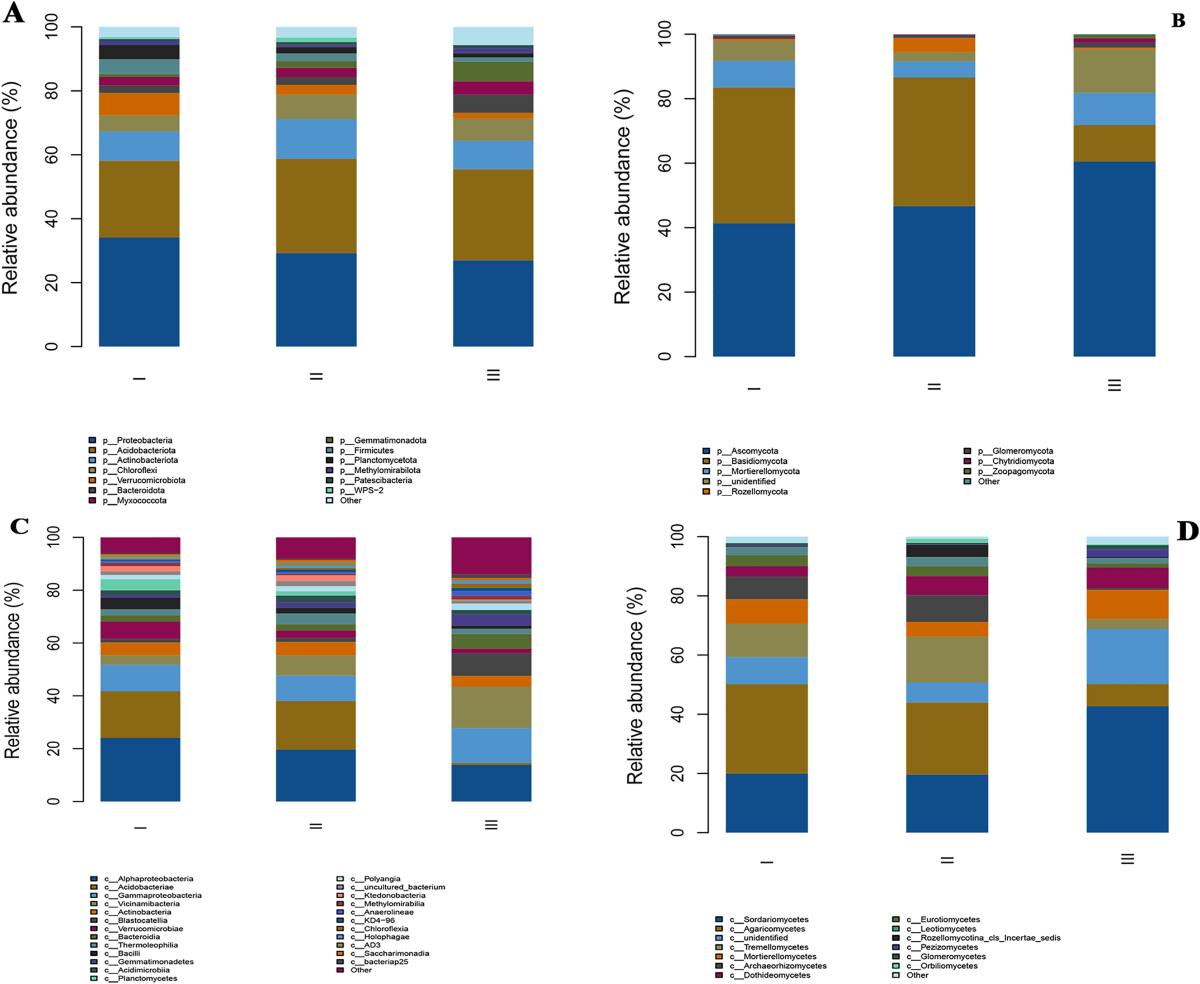
The relative abundances of bacterial and fungi communities at the phylum level (A and B) and the class level (C and D) in the different samples. All phyla and classes with a relative abundance were greater than 1%. The members with a degree of <1% were indicated as “other”. I, Wild Qizhu rhizosphere soil sample; II, wild Shezhu rhizosphere soil sample; III, Bozhu rhizosphere soil sample. (A & C) Bacteria; (B & D) fungi.

Through high-throughput sequencing, a total of 16 phyla, 29 classes, 49 orders, 51 families, and 39 genera of bacteria and eight phyla, 17 classes, 37 orders, 65 families, and 71 genera of fungi were identified in the rhizosphere soil of wild genuine Qizhu and Shezhu samples and artificially cultivated Bozhu samples (Fig. 3). The microbial communities in both the wild and artificial cultivated soil samples were extremely species-rich, but there were some differences in their structure and the relative abundances of Microorganism taxa.

At the phylum level, the main bacterial phyla in the rhizosphere samples of Qizhu, Shezhu, and Bozhu were *Proteobacteria*, *Acidobacteriota*, *Actinobacteria*, *Chloroflexi*, *Verrucomicrobiota*, *Bacteroidetes*, *Myxomycota*, *Firmicutes*, and *Planctomycetota* (Fig. 3A). *Proteobacteria* and *Acidobacteriota* were the dominant groups in the rhizosphere samples of RAM from three different habitats, accounting for more than 50%. However, the abundances of *Proteobacteria* and *Acidobacteriota* were significantly different among the three samples. The relative abundance of *Proteobacteria* in the wild Qizhu sample was 34.1%, which was higher than that in Shezhu and Bozhu (29.3% and 27.0%) (*P* < 0.05). The relative abundances of *Acidobacteriota* were 29.4% and 28.5% in Shezhu and Bozhu respectively, which were higher than those in Qizhu (24.0%). In addition, the relative abundances of *Verrucomicrobiota*, and *Planctomycetota* in Qizhu were higher than those in Shezhu and Bozhu (*P* < 0.05). In Shezhu, the numbers of *Actinobacteriota* and *Chloroflexi* were significantly greater than those in Qizhu and Bozhu. The relative abundance of *Bacteroidetes* in the rhizosphere soil of the artificially cultivated Bozhu sample was 5.7%, which was higher than that of the rhizosphere soil samples of Qizhu and Shezhu (2.4%). Among the fungi, A*scomycota*, *Basidiomycota*, *Mortierellmycota*, *Rozellomycota*, *Glomeromycota*, and *Chytridiomycota* were the main fungal phyla in the rhizosphere samples of Qizhu, Shezhu, and Bozhu (Fig. 3B). Among them, *Basidiomycota* and *Ascomycota* were the dominant groups shared by the three samples, with relatively high relative abundance, accounting for more than 80%. The relative abundances of *Basidiomycota* in Qizhu and Shezhu were similar but significantly higher than those in the rhizosphere soil sample of Bozhu. In Bozhu, the relative abundance of *Ascomycota* (60.5%) was significantly higher than that in Qizhu and Shezhu (41.3% and 46.7%) (*P* < 0.05). In addition, the relative abundance of *Rozellomycota* in Shezhu was higher than that in Qizhu and Bozhu rhizosphere soil samples (*P* < 0.05).

At the class level, for bacterial groups, *Alphaproteobacteria* and *Acidobacteriae* were the dominant groups shared by Qizhu, Shezhu, and Bozhu, accounting for more than 40%. *Alphaproteobacteria* had the highest relative abundance (24%) in Qizhu, which was higher than that in Shezhu and Bozhu (19.6% and 13.8%, respectively). The relative abundances of *Acidobacteriae* in Qizhu and Shezhu were 17.6% and 18.4%, respectively, which were significantly higher than that in Bozhu (0.8%) (*P* < 0.05). *Vicinamibacteria* was the dominant group in Bozhu, and its relative abundance was significantly higher than that in Qizhu and Shezhu. In addition, the relative abundance of *Verrucomicrobiae* in Qizhu was higher than that in the rhizosphere samples of Shezhu and artificially cultivated of Bozhu (*P* < 0.05). In Shezhu, the number of *Thermoleophilia* was higher than that in Qizhu and Bozhu. The relative abundance of *Gammaproteobacteria* in the artificially cultivated Bozhu sample was 13.1%, higher than that in Qizhu and Shezhu rhizosphere soil samples (Fig. 3C). Among the fungal groups, *Sordariomycetes* and *Agaricomycetes* were the most abundant in Qizhu and Shezhu, accounting for about 50% of Qizhu and 40% of Shezhu. The relative abundance of *Sordariomycetes* was the highest (42.7%) in the artificially cultivated Bozhu sample, which was significantly higher than that in Qizhu and Shezhu (20% and 19.6%) (*P* < 0.05). At the same time, the relative abundances of *Tremellomycetes* and *Archaeorhizomycetes* in the rhizosphere soil samples of Shezhu were significantly higher than those in the rhizosphere soil samples of Qizhu and Bozhu (Fig. 3D).

**Figure 4:**
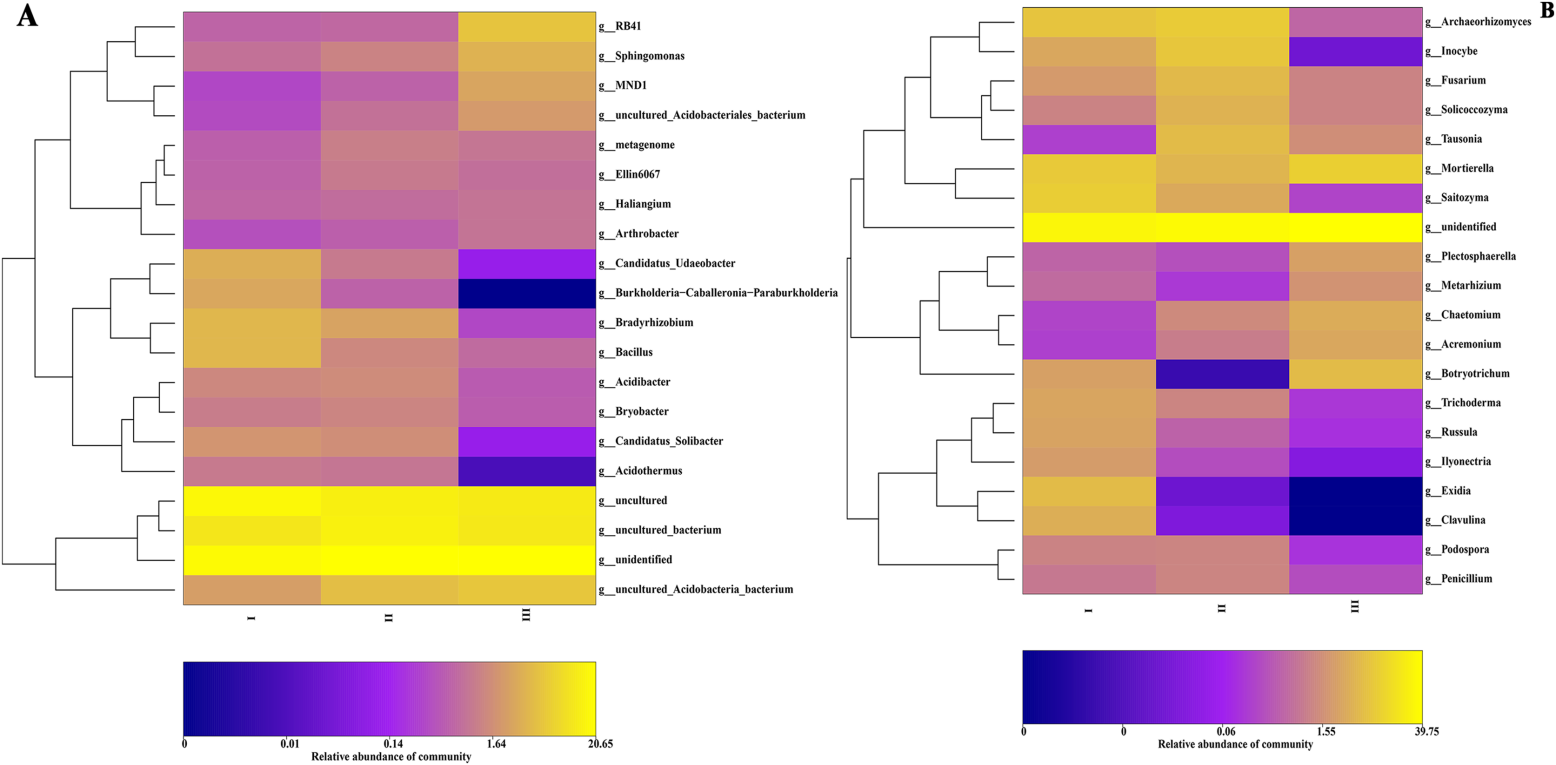
The heat map of community composition of bacteria and fungi in rhizosphere soil of wild Qizhu, Shezhu and cultivated Bozhu at genus level. (A) Bacteria; (B) fungi. In the diagram, red represents richer genera in the corresponding samples, and blue represents less abundant genera. I, Wild Qizhu rhizosphere soil sample; II, Shezhu rhizosphere soil sample; III, Bozhu rhizosphere soil sample.

The community composition structure at each taxonomic level was clustered according to the abundance distribution of the taxa and the degree of similarity between samples. According to the clustering results, the taxa and samples were sorted separately and represented using a heat map. Through clustering, high-abundance and low-abundance taxa were generally distinguished, and a color gradient was used to reflect the similarity of community composition between samples. At the genus level, the most abundant 20 genera of bacteria and 16 genera of fungi in 16 samples from three producing areas were analyzed by a heat map. Bacterial community abundance analysis showed (Fig. 4A) that the dominant bacteria in Qizhu were Bradyrhizobium, Bacillus, Candidatus-udaeobacter, Candidatus-solibacter, and Burkholderia-caballeronia-paraburkholderia, accounting for 4.2%, 4.1%, 3.4%, 2.0%, and 2.9% respectively. These relative abundances were significantly higher than those in the rhizosphere soil samples of Shezhu and Bozhu.

Among them, the relative abundances of Bradyrhizobium, Bacillus, and Candidatus-udaeobacter were 14, 5.3, and 34 times higher, respectively, than those of Bozhu rhizosphere soil (*P* < 0.05). The dominant bacteria in the Shezhu sample were Bradyrhizobium, Candidatus-solibacter, Acidibacter, and Bryobacter, with relative abundances of 2.6%, 1.7%, 1.6%, and 1.4% respectively, which were significantly higher than those in the rhizosphere soil of Bozhu. Meanwhile, the relative abundance of Bryobacter was also higher than that of Qizhu. RB41, Sphingomonas and MND1 were the dominant genera in the Bozhu rhizosphere soil sample, and their relative abundances were 5.6%, 3.7%, and 2.7%, respectively, representing significantly higher values than those in Qizhu and Shezhu rhizosphere soil samples. The relative abundances of RB41 and MND1 in the Qizhu and Shezhu samples were less than 1% (*P* < 0.05). It was worth noting that Burkholderia-caballeronia-paraburkholderia had a high relative abundance (2.9%) in Qizhu samples, which was 4.8 times higher than that in Shezhu rhizosphere soil samples, and there was almost no enrichment of this genus in the Bozhu sample (*P* < 0.05). For fungi (Fig. 4B), among the rhizosphere soil sample of Qizhu, the dominant genera were Saitozyma, Exidia, Clavulina, and Russula, and their relative abundances were 9.4%, 5.8%, 3.8%, and 2.8% respectively, which were higher than those of the rhizosphere soil samples of Sezhu and Bozhu (*P* < 0.05). In Qizhu, Saitozyma enrichment was 2.8 times that of Shezhu and 47 times that of Bozhu, while Russula enrichment was 5.6 times that of Shezhu, and 28 times that of Bozhu. Additionally, Exidia and Clavulina had a high abundance in Qizhu, and their relative abundances in Shezhu were less than 0.1%, while these two bacteria were not detected in Bozhu. Simultaneously, Archaeo, Inocybe, Fusarium, Tausonia, and Solicoccozyma were the dominant genera in the rhizosphere soil sample of Shezhu, and their relative abundances were 9.0%, 8.0%, 5.4%, 5.7%, and 4.4%, respectively, representing higher values than those in rhizosphere soil samples of Qizhu and Bozhu (*P* < 0.05). In the rhizosphere soil sample of Bozhu, Mortierella, Botryotrichum, Chaetomium, Acremonium, Trichoderma, and Plectosphaerella were dominant, and their relative abundances were 9.8%, 5.6%, 3.6%, 3.2%, 3.2%, and 2.6%, respectively, representing higher values than those in Qizhu and Shezhu, but the relative abundances of other genera were lower. Therefore, the relative abundances of bacteria and fungi in rhizosphere soils of wild Qizhu and Shezhu and cultivated Bozhu were significantly different at the genus level.

### Beta-diversity analysis of the rhizosphere microbial community of RAM from three production areas

**Figure 5:**
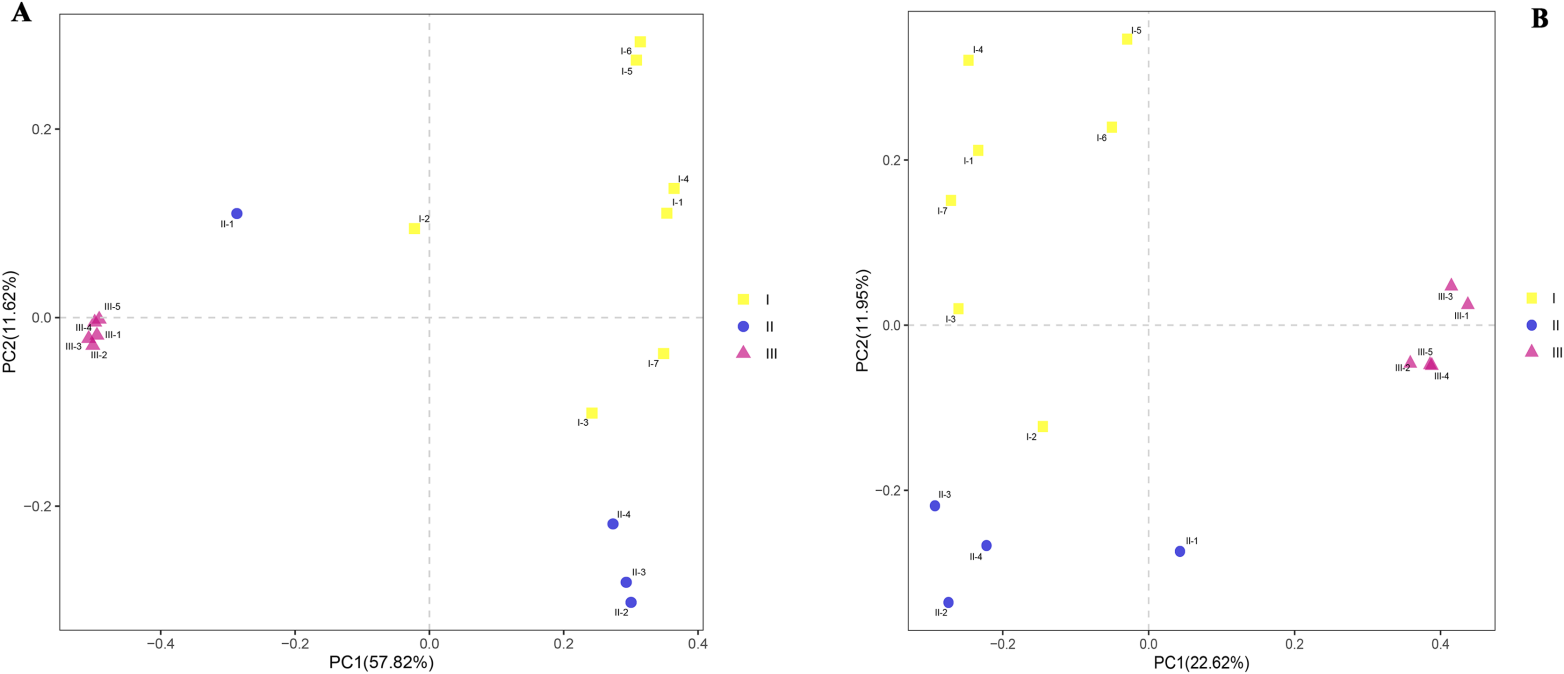
Beta diversity. (A and B) Bray-Curtis PCoA analysis. For the bacteria and fungal communities in rhizospheric soil from wild and reintroduced *M. sinica* at the OTU level using the unweighted UniFrac method. I1-I7, Wild Qizhu rhizosphere soil sample; II1-II4, Shezhu rhizosphere soil sample; III1-III5, Bozhu rhizosphere soil sample. (A) Bacteria; (B) fungi.

To observe similaries and differences between samples, principal component analyses (PCoA) were carried out on the differences between the three soils. PCoA revealed substantial inter- and intra- group variation in microbial communities depending on the samples (Figs. 5A  and 5B). Principal component 1 (PC1) interpreted both inter-group and intra-group variations, while principal component 2 (PC2) mainly explained the intra-group variation. The microbial community structures (bacteria and fungi) of rhizosphere samples of Qizhu and Shezhu had relatively great intra-group variation, but there was relatively small intra-group variation in the compositions of microbial communities in Bozhu. Moreover, the bacterial community structures in Qizhu and Shezhu differed from those in Bozhu. There were clear seen that there were significant differences in the structure of bacteria and fungi in rhizosphere soil between wild and cultivated RAM.

### Content analysis of polysaccharides, atractylone, and lactone I, II, and III

Powders of RAM samples from the Qimen, Shexian, and Bozhou cities of the Anhui Province were obtained. Three portions of each sample were used, and three needles were repeated for each portion. The contents of polysaccharides, atractylone, and lactone I, II, and III from three samples were determined and shown in Table 2. The results showed that the contents of lactone I, II, III, and atractylone were significantly different among the three habitats (*P* < 0.05). The contents of lactone I, II, III, atractylone, and polysaccharides were the highest in Shezhu, which had lactone I contents that were 1.54 and 1.82 times higher than those in Qizhu and Bozhu, respectively. The contents of lactone II in Shezhu were 1.38 and 2.53 times higher than those in Qizhu and Bozhu, respectively. The contents of lactone III in Shezhu were 1.46 times and 1.89 times higher than that in Qizhu and Bozhu, respectively. Atractylone and polysaccharide contents in Shezhu were 1.13 times and 1.15 times higher than those in Qizhu, and 3.14 times and 1.12 times higher than those in Bozhu. The contents of atractylone and lactone I, II, and III in Qizhu were significantly higher than those in Bozhu (*P* < 0.05). Polysaccharide contents in Qizhu and Bozhu showed had no significant differences (*P* > 0.05).

**Table 2:** Contents of Lactone I, Lactone II, Lactone III, atractylone and polysaccharides in different origins (*n* = 3).

Chemical composition (mg/g)	Qizhu	Shezhu	Bozhu
Lactone I	0.26 ± 0.0012b	0.40 ± 0.0073a	0.22 ± 0.0019c
Lactone II	0.55 ± 0.0033b	0.76 ± 0.0011a	0.30 ± 0.0032c
Lactone III	0.71 ± 0.0200b	1.04 ± 0.0022a	0.55 ± 0.0080c
Atractylone	15.93 ± 0.0150b	18.04 ± 0.0320a	5.74 ± 0.0370c
Polysaccharides	534.76 ± 19.98b	616.18 ± 23.9000a	551.92 ± 8.3200b

**Note:**

Lowercase letters indicate significant differences between different origins (*P* < 0.05).

### Correlation between rhizosphere microorganisms and the accumulation of polysaccharides, atractylone, and lactones in RAM

**Figure 6:**
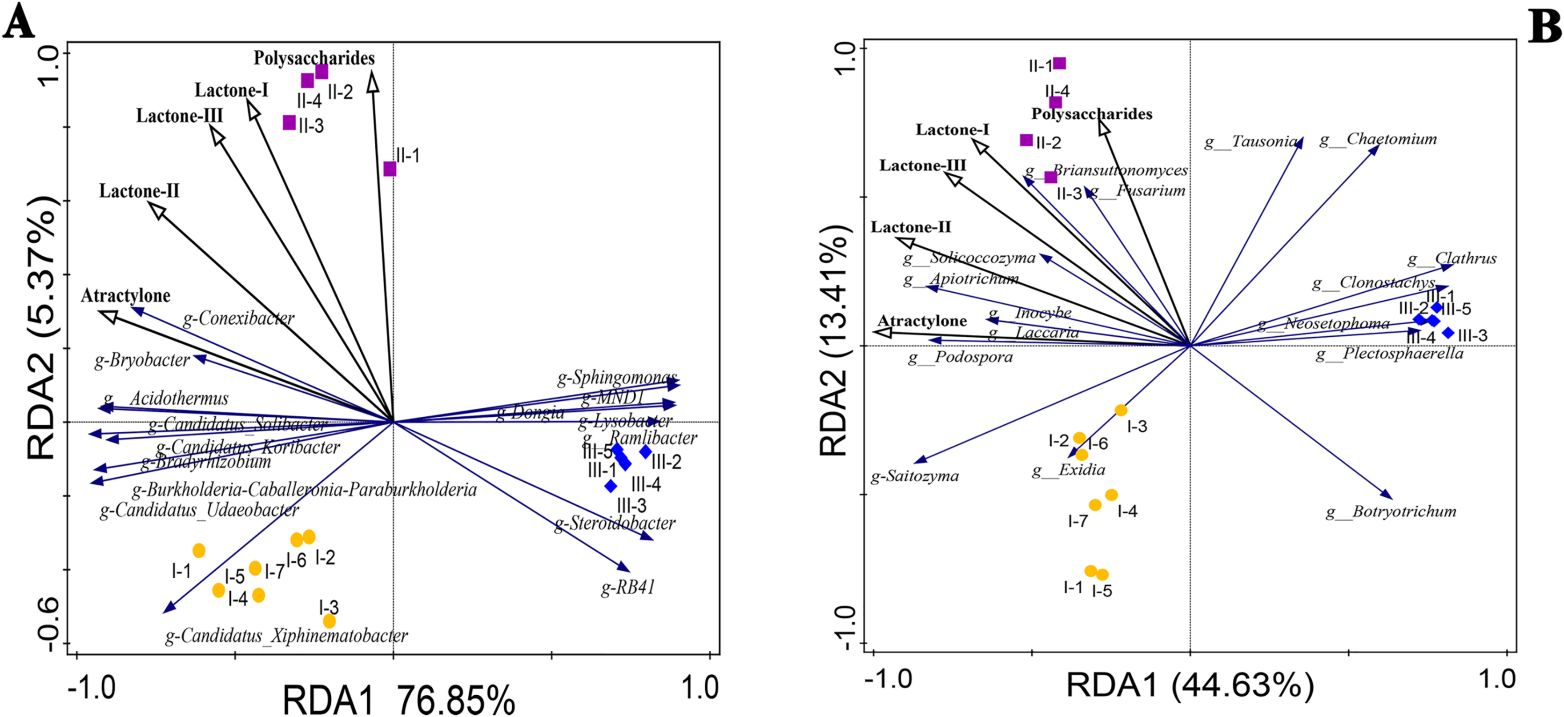
Redundancy analysis (RDA) plots of the bacterial (A) and fungal (B) communities with respect to environmental variables in the root zone of RAM. Points with different shapes and colors represent different samples. The closer the distance between the two points in the figure is, the smaller the difference in community structure between the two samples is, and the higher the similarity is; the black ray represents different environmental factors (explanatory variables), and the length of the ray represents the degree of influence of environmental factors on species data (explanatory amount); the angle between environmental factor rays represents positive and negative correlation (acute angle: positive correlation; obtuse angle: negative correlation; right angle: no correlation). The vertical projection from the sample point to the ray of the environmental factor, the closer the projection point is to the ray direction of the environmental factor, the greater the influence of the environmental factor on the sample community structure; blue rays represent different species (response variables), and the direction in which the rays point indicates the direction in which the abundance of that species increases. The starting point of the species arrow indicates the average position of species abundance. If the quadrat is on the backward extension line of the arrow, it means that the abundance of the species in the quadrat is less than the average value. Otherwise, it is greater than the average. The angle between species and species and between species and environmental factor rays represents positive and negative correlations (acute angle: positive correlation; obtuse angle: negative correlation; right angle: no correlation). I1-I7, Wild Qizhu rhizosphere soil sample; II1-II4, Shezhu rhizosphere soil sample; III1-III5, Bozhu rhizosphere soil sample).

The correlation coefficients between the accumulation of polysaccharides, atractylone, and lactones in RAM and the top 16 bacteria and fungi at the genus level were calculated using redundancy analysis (RDA) and shown in Fig. 6. Among them, the microbial community composition at the genus level was the response variable and the chemical components were the explanatory variables. We used Pearson or Spearman’s correlation analysis to show significant *P* values for explanatory variables. Among the *P* values of explanatory variables, those of ateactylone, lactone II, and lactone III in bacteria were 0.002, 0.002, and 0.04, respectively, denoting significant differences. There were no significant differences between lactone I and polysaccharide enrichment among bacteria (*P* > 0.05). Among fungi, there were significant differences in atractylone and lactone-II (*P* < 0.05), while there were no significant differences in the other three components (*P* > 0.05). The correlation between explanatory variables and responsibility variables can be combined with the second point above, and the correlation between explanatory variables and response variables can be analyzed according to the angle relationship between explanatory variables and response variables in Fig. 6 RDA. *Conexibacter*, *Bryobacter*, *Acidothermus*, *Candidatus-Solibacter*, *Candidatus-Koribacter*, *Bradyrhizobium*, *Burkholderia-Caballeronia-Paraburkholderia*, *Candidatus-Udaeobacter*, and *Candidatus-Xiphinematobacter* in bacteria were positively correlated with atractylone and lactone II contents (Fig. 6A). Except for *Candidatus-Xiphinematobacter*, the above bacterial genera were positively correlated with lactone I and III compounds. In addition, polysaccharide content was positively correlated with *Conexibacter*, *Bryobacter*, *Sphingomonas*, and *MNDI*. According to the RDA statistical data table of bacterial strains in Table 3, except for *Sphingomonas* and M*NDI*, the contents of these bacteria in Qizhu and Shezhu were higher than those in artificially cultivated RAM samples. Meanwhile, *RB41*, *Steroidobacter*, *Aamlibacter*, and *Lysobacter* were significantly negatively correlated with the accumulation of these five compounds. In Table 3, the relative abundance of these four strains in Bozhou was the highest, being higher than that of Qizhu and Shezhu rhizosphere soil samples. Among fungi (Fig. 6B), *Briansuttonomyces*, *Apiotrichum*, *Podospora*, *Fusarium*, *Solicoccozyma*, *Inolyma*, and *Laccuria* were all positively correlated with the accumulation of the five compounds. *Saitozyma* was positively correlated with atractylone, lactone I, II, and III. *Tausonia* and *Chaeotomium* were positively correlated with polysaccharides contents. At the same time, in the RDA statistical data table of fungal strains in Table 4, the relative abundance of these genera in Shezhu was higher than that in Qizhu and Bozhu, and their relative abundance in Bozhu samples was the lowest. On the contrary, *Botryotrichum*, *Plectosphaerella*, and *Neosetophoma* were negatively correlated with the content of the above five compounds. As shown In Table 4, the contents of these three fungi in Bozhu were the highest, being higher than those in Qizhu and Shezhu rhizosphere soil. These results showed that rhizosphere bacteria and fungi were involved in the accumulation of polysaccharides, atractylone, and lactones.

**Table 3:** RDA statistics of different bacterial strains.

Responsible variable	I	II	III
g-Candidatus_Udaeobacter	0.0341	0.0105	0.0011
g-Bradyrhizobium	0.0421	0.0264	0.0035
g-Sphingomonas	0.0088	0.0130	0.0365
g-MND1	0.0034	0.0062	0.0271
g-Dongia	0.0012	0.0017	0.0116
g-Conexibacter	0.0036	0.0084	0.0001
g-Lysobacter	0.0006	0.0010	0.0072
g-Steroidobacter	0.0012	0.0008	0.0108
g-Candidatus_Xiphinematobacter	0.0146	0.0028	0.0005
g-Candidatus_Koribacter	0.0089	0.0049	0.0001
g-RB41	0.0065	0.0071	0.0559
g-Candidatus_Solibacter	0.0196	0.0166	0.0011
g__Ramlibacter	0.0012	0.0018	0.0071
g__Acidothermus	0.0107	0.0098	0.0001
g-Bryobacter	0.0115	0.0139	0.0055
g-Burkholderia-Caballeronia-Paraburkholderia	0.0285	0.0063	0

**Note:**

Each number multiplied by one hundred percent is its relative abundance percentage. I, II, and III represent rhizosphere soil of Qizhu, Shezhu, and Bozhu, respectively.

**Table 4:** RDA statistics of different bacterial strains.

Responsible variable	I	II	III
g-Saitozyma	0.0940	0.0335	0.0019
g__Clonostachys	0.00002	0.0003	0.0189
g__Exidia	0.0583	0.00009	0
g__Clathrus	0.0001	0.0004	0.0108
g__Apiotrichum	0.0023	0.0187	0.00001
g__Neosetophoma	0.00004	0.00005	0.0214
g__Chaetomium	0.00006	0	0
g__Podospora	0.0116	0.0121	0.0011
g__Briansuttonomyces	0.0004	0.0117	0.00003
g__Plectosphaerella	0.00478	0.0027	0.0259
g__Inocybe	0.0325	0.0801	0.0001
g__Botryotrichum	0.0259	0.00001	0.0561
g__Laccaria	0.00002	0.0185	0
g__Tausonia	0.0017	0.0565	0.0164
g__Solicoccozyma	0.01182	0.0440	0.0118
g__Fusarium	0.0226	0.0543	0.0114

**Note:**

Each number multiplied by one hundred percent is its relative abundance percentage. I, II, and III represent rhizosphere soil of Qizhu, Shezhu, and Bozhu, respectively.

### Function prediction of bacteria and fungi in rhizosphere soil of wild and cultivated RAM

**Figure 7:**
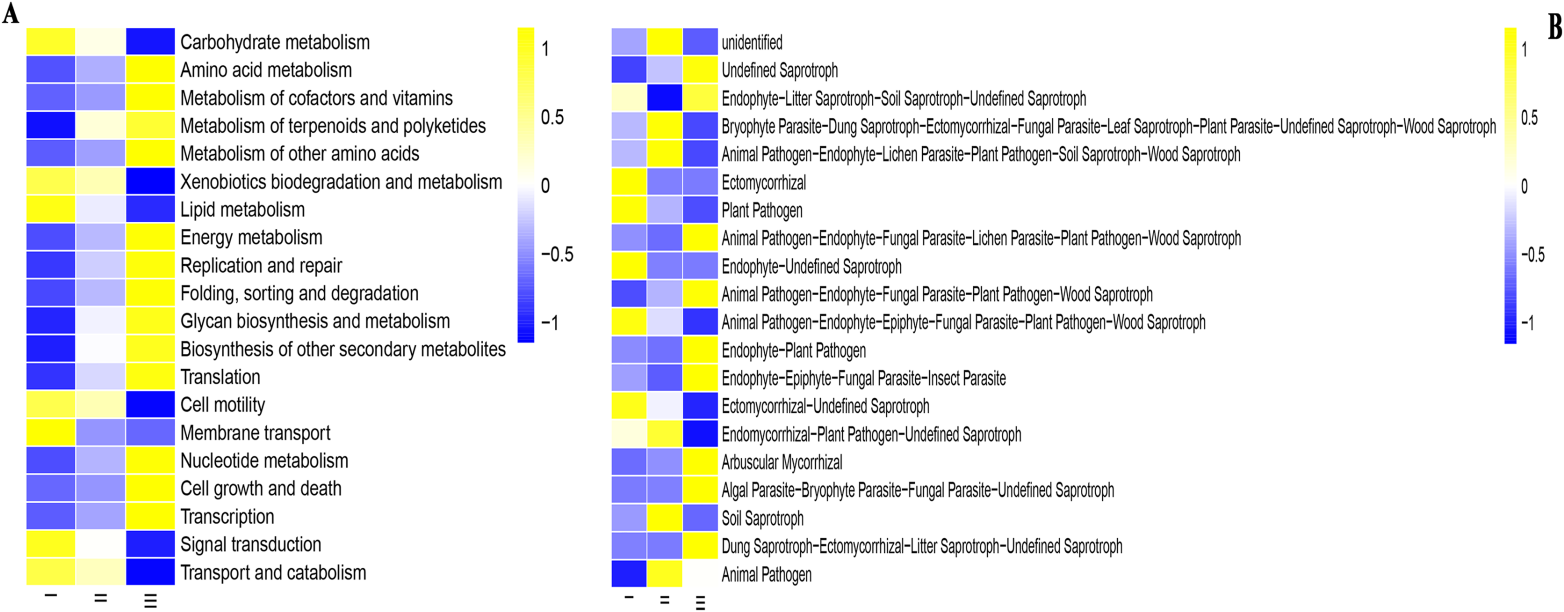
Functions of bacteria and fungi in rhizosphere soil of wild and cultivated RAM. (A) Bacteria; (B) fungi. I, Wild Qizhu rhizosphere soil sample. II, Shezhu rhizosphere soil sample. III, Bozhu rhizosphere soil sample.

PICRUSt software was used to predict the function of microbial communities (Figs. 7A and 7B). For the convenience of the display, only the pathways with the most enrichment in the samples were shown. In the PICRUSt method, Qizhu was more enriched in the following functions: Carbohydrate metabolism, Xenobiotics biodegradation and metabolism, Lipid metabolism, Cell motility, Membrane transport, and Signal transduction. In Shezhu, the abundance of these functional groups was not very prominent, basically between Qizhu and Bozhu rhizosphere soil samples. Bozhu has relatively rich functions in Amino acid metabolism, Metabolism of cofactors and vitamins, Metabolism of terpenoids and polyketides, Energy metabolism, Replication and repair, Nucleotide metabolism, *etc* (Fig. 7A). However, there was little difference in abundance between the various functional groups of the three samples.

The sequences of the fungi isolated from the wild and reintroduced rhizospheric soil were compared with those in the FUNGuild database to predict and analyze differences in their functions (Chen et al., 2020; Del Pilar Martínez-Diz et al., 2019). Figure 7B was divided into different functional groups according to the fungal groups in wild and cultivated rhizosphere soils. A total of 20 functional groups were identified in this study, including Animal Pathogen, Plant Pathogen, Ectomycorrhizal, Dung Saprotroph, Undefined Saprotroph, Dung Saprotroph, Wood Saprotroph, Plant Saprotroph, Soil Saprotroph, Endomycorrhizal, Endophyte, Arbuscular Mycorrhizal, Fungal Parasite, Lichenizeda and Unknown. The results showed that there were many fungal groups with an unidentified trophic mode (defined as having an unknown trophic mode) in wild and cultivated rhizosphere soils. Both the wild and reintroduced rhizospheric soils hosted many unknown groups, whose relative abundance in the Qizhu rhizospheric soil was the highest (53%), being higher than those in the rhizosphere soil of Shezhu and cultivated Bozhu (45.9% and 44%). The results also showed that except for unidentified, the relative abundance of Ectomycorrhizal species in the rhizosphere soil of wild Qizhu was the highest, with a relative abundance of 7.8%, which was significantly higher than those of the rhizosphere soil of Shzhu and Bozhu (0.6% and 0.5%, respectively) (*P* < 0.05). The second was Endophyte-Undefined Saprotroph, with a relative abundance of 6.3%, which was also significantly higher than those of Shezhu and Bozhu rhizosphere soils (0.1% and 0.001%, respectively) (*P* < 0.05). The relative abundance of Bryophyte Parasite-Dung Saprotroph-Ectomycorrhizal-Fungal Parasite-Leaf-Saprotroph-Plant Parasite-Undefined Saprotroph-Wood Saprotroph in the rhizosphere soil of Shezhu high (11.2%), being significantly higher than those in Qizhu and Bozhu rhizosphere soils (6.2% and 4.4%, respectively) (*P* < 0.05). Followed by Animal Pathogen-Endophyte-Lichen Parasite-Plant Pathogen-Soil Saprotroph-Wood Saprotroph 5.8%, higher than Qizhu and Bozhu samples (2.4% and 1.2%). For the rhizosphere soil of Bozhu, the relative abundance of Undefined Saprotroph was high (17.1%), being significantly higher than that of wild Qizhu and Shezhu (8.5% and 10.9%, respectively) (*P* < 0.05). The second is Endophyte-Litter Saprotroph-Soil Saprotroph-Undefined Saprotroph, with a relative abundance of 10.5%, which is higher than those of Qizhu and Shezhu samples (8.8% and 0.5%, respectively).

## Discussion

Plants can shape and recruit protective microorganisms from soil microbial communities to form rhizosphere microbial communities (Berendsen, Pieterse & Bakker, 2012), These microbial communities are key factors influencing the relationships between plants, soil, and microbes; are essential for plant health; and are the most active areas of material and energy exchange (Li et al., 2018). Sequence analysis identified 16 bacterial phyla in were identified from the rhizosphere soil of RAM. Among them, *Proteobacteria* was the dominant phyla, accounting for 27.0–34.1% of the sample sequences, followed by *Acidobacteriota* (24.0–29.4%). Studies had shown that *Proteobacteria* and *Acidobacteriota* were common microbial communities in other plants and soils (Chen et al., 2019; Jackson et al., 2013). Among them, *Proteobacteria* was the dominant bacteria in Qizhu. It had been reported that *Proteobacteria* play an important role in natural processes, having the ability to fix heavy metals, solubilize phosphorus, and degrade hydrocarbons in natural processes and having a strong antifungal effect on the plant pathogen *Fusarium oxysporum*. They were also found to have the ability to produce siderophores and use toxic pollutants to promote plant growth (Drzewiecka, 2016) and had potential applications in the treatment of wastewater, improving the tolerance to pollutants and improving soil environments (Lin et al., 2019; Kragelund et al., 2007). It has also been revealed that *Thermoacid bacteria* in *Acidobacteriota* play an important role in plant processes and could promote plant growth, which was closely related to the accumulation of active ingredients (Kalam et al., 2020).

In terms of fungi, our results showed that *Ascomycota*, *Basidiomycota*, *Mortierellomycota*, unclassified, and *Rozellomycota* were the dominant groups in the rhizosphere soils of wild genuine Qizhu, Shezhu, and artificially cultivated Bozhu samples. Studies had shown that fungi were important decomposers in ecosystems and could effectively decompose cellulose and lignin (Tan et al., 2021). Among them, *Ascomycota* and *Basidiomycota* were important fungal phyla in most soils (Hussain et al., 2011; Wallenstein, McMahon & Schimel, 2007), and also the main dominant fungi in rhizosphere soil. The members of these two phyla could participate in the carbon cycle by degrading organic matter (Unterseher, Peršoh & Schnittler, 2013; Purahong et al., 2016). In this study, we observed significant differences in the relative abundance of *Ascomycota* and *Basidiomycota* in our samples. In particular, the relative abundance of *Ascomycota* in Bozhu rhizosphere soil (60.5%) was higher than that in wild Qizhu rhizosphere soil (41.3%) and Shezhu rhizosphere soil (46.7%) (*P* < 0.05). Members of *Ascomycota* have been found to play an important role in decomposing plant residues and degrading OM in soil (Frey et al., 2004). Shen et al. (2020) also reported that the relative abundance of *Ascomycota* in cultivated rhizosphere soil was higher than that in wild rhizosphere soil. The reason may be that the degree of degradation of organic matter in cultivated rhizosphere soil is higher than that in wild rhizosphere soil, which leads to the increase of in *Ascomycota* abundance to levels higher than those in wild rhizosphere soil. During the collection, it was found that there were many fallen leaves and litter in the place where wild genuine Qizhu grew, while the place where Bozhu grew, with only a small amount of litter. Interestingly, the analysis results also showed that the relative abundance of *Basidiomycota* in the rhizosphere soil of wild Qizhu (42.2%) was significantly higher than that in the rhizosphere soil of Bozhu (11.3%) (*P* < 0.05). *Basidiomycota* could effectively decompose lignocellulose, indicating that the members of the classification unit could effectively decompose a large number of leaves and litter (Shen et al., 2020). It can also be seen that plant rhizosphere microbial communities play an important role in plant growth and health.

At the genus level, according to the heat map (Fig. 4), *Bacillus* and *Russula* were dominant in the rhizosphere soil samples of Qizhu, with their levels being significantly higher than those in Bozhu samples (*P* < 0.05). On the contrary, the relative abundance of *Mortierella* in Bozhu was higher than that in Qizhu and Shezhu. The growth environment of wild RAM is harsh, which poses a certain challenge to its growth. This is one of the reasons why wild RAM is on the verge of extinction. It had been researched that *Bacillus* was not only a dominant bacterium but also a beneficial microorganism, having antagonistic and growth-promoting effects and promoting crop growth (Lou et al., 2018). Pitzschke (2016) also showed that *Bacillus* potentially manipulates the host’s redox status and contributes to overcoming a critical period in development and seedling establishment due to its high catalase activities and superoxide contents. Among fungi, *Mortierella* are very common in soil, having a similar role to that of Ascomycota. For example, they can promote the absorption of minerals by plant roots and inhibit pathogenic microorganisms (Klingen, Eilenberg & Meadow, 2002). These results are similar to those reported in the literature on *Ascomycota* (Shen et al., 2020). We speculate that the degradation of organic matter in cultivated rhizosphere soil being higher than that in wild rhizosphere soil results in an increase in the abundance of *Mortierella* and *Ascomycota*, leading to higher levels than those in wild rhizosphere soil.

Previous studies had shown that various bioactive components originate from microorganisms or interact with the host through microorganisms (Bulgarelli et al., 2012). The correlation between rhizosphere microorganisms and the accumulation of polysaccharides, atractylone, and lactone compounds was considered in this study. According to the accumulation of atractylone, polysaccharides and lactone, the results showed that rhizosphere microorganisms played an important role. The rhizosphere microbial abundance was different between the wild and cultivated RAM samples. Among the bacteria, the relative abundances of *Conexibacter*, *Bryobacter*, *Acidothermus*, *Candidatus-Solibacter*, *Candidatus-Koribacter*, *Bradyrhizobium*, *Burkholderia-Caballeronia-Paburkholderia*, *Candidatus-Udaeobacter*, *Candidatus-Xiphinematobacter* were higher in Qizhu and Shezhu. Their relatives abundance in Qizhu and Shezhu was 0.36%, 1.15%, 1.07%, 1.96%, 0.89%, 4.21%, 2.85%, 3.41%, 1.4% and 0.84%, 1.39%, 0.98%, 1.66%, 0.49%, 2.64%, 0.63%, 1.05%, 0.28%, respectively, while those in Bozhu was 0.01%, 0.55%, 0.01%, 0.11%, 0.01%, 0.35%, 0%, 0.11%, 0.05%, respectively. Among the fungi: The relative abundance of *Briansuttonomyces, Apiotrichum, Podospora, Fusarium, Solicoccozyma, Inolyma, Laccuria* in Shezhu was 1.17%, 1.87%, 1.21%, 5.43%, 4.40%, 8.01%, 1.85%, higher than that in Qizhu (0.04%, 0.23%, 1.16%, 2.26%, 1.18%, 3.25%, 0.002%) and Bozhu (0.003%, 0.001%, 0.11%, 1.14%, 1.18%, 0.001%, 0%). Chen et al. (2022) found that *Conexibacter* is a dominant carbon-fixing bacteria, which is positively correlated with root and plant dry weight. Studies have shown that *Candidatus-Solibacter* and *Bryobacter* are functional bacteria related to the transformation of substances in soil. They can decompose organic matter, promote soil carbon cycle and nitrogen fixation, and play a positive role in improving the soil environment (Fan et al., 2021). In addition, Cheng et al. (2022) also found that *Solicoccozyma* had the strongest correlation with some metabolites, indicating that this microorganism may be involved in the synthesis of most metabolites in soil. These microorganisms were positively correlated with the accumulation of the main active components of RAM. This may also be one of the key reasons for the accumulation of chemical components in wild Qizhu and Shezhu.

Soil bacterial communities may be important for plants to maintain basic functions. In studying the predictive function of this community, we found that its carbohydrate and amino acid metabolism were predicted to be high. Carbohydrates account for over 50% of plant dry weight, are important organic matter, and can provide nutrients to promote plant root growth (Naylor & Coleman-Derr, 2018). Amino acids such as glutamine, proline and glycine betaine can enhance plant growth, increase dry matter accumulation, and promote plant yield. Our data also show that the abundance of bacterial membrane transport in Qizhu is high. According to previous studies, the membrane transport of bacteria can improve the salt and drought tolerance of plants by regulating the Na^+^/K^+^ ratio and H^+^-ATPase of the plasma membrane, enhancing the growth and accumulation of beneficial components of plants in the form of plant growth-promoting bacteria. According to how they obtain nutrition, fungi can be divided into three trophic modes: pathotroph, saprotroph, and symbiotroph (Nguyen et al., 2016). Saprotrophic fungi obtain nutrients by decomposing dead host cells, symbiotroph obtain nutrients by exchanging resources with host cells, and pathotroph acquire nutrients by harming host cells (Delgado-Baquerizo et al., 2021).

The results showed that the fungi in wild rhizosphere soil were mainly saprophytic, and the relative abundance of ectomycorrhizal fungi was also higher in Qizhu. The fungi in the rhizosphere soil of artificially cultivated RAM are mainly saprophytic bacteria, followed by symbiotic bacteria. In addition, previous studies have shown that *Russula* accounted for the vast majority of *Russulaceae*. This genus has significant economic, edible, and medicinal value and may have symbiotic ectomycorrhizal relationships with trees (Tan et al., 2013). According to the results of the heat map and functional prediction, *Russula* dominated the rhizosphere soil samples of Qizhu, and its relative abundance in Bozhu was less than 0.1%. Therefore, the structure and function of fungal communities were consistent, indicating that our results are reliable. The wild rhizosphere soil fungi mainly obtain nutrients by decomposing litter, while the reintroduced rhizosphere soil fungi predominantly acquire nutrients by exchanging substances with plant cells and decomposing dead host cells. The statistical analysis of functional fungal groups showed that the relative abundance of plant pathogenic bacteria in the rhizosphere soil of wild plants was lower than that in the rhizosphere soil of reintroduced plants, indicating that there were many antagonistic microorganisms in the rhizosphere soil of wild plants. Biological control microorganisms can be obtained by culture-dependent methods to promote the healthy growth of plants and enhance the accumulation of effective components.

These results have potential implications for research on the RAM rhizosphere. Only by deeply understanding the potential functions of the rhizosphere microorganisms and the synthesis of the main chemical components of RAM, the relationship between the rhizosphere microorganisms and the accumulation of lactones, polysaccharides, and atractylone could be understood. In such experiment, we explored the effect of rhizosphere microorganisms on the effective components of RAM. The control soil sample (non-rhizosphere soil sample) from each sampling site should also be included for comparison with the rhizosphere soil sample in future work. Overall, in the long run, this information could help screen beneficial probiotics as biofertilizers to increase yields and medicinal values and promote the sustainable development of RAM.

## Conclusions

The composition, structure, diversity, and function of bacterial and fungal communities in the rhizosphere of wild and cultivated RAM, as well as the correlation between the main chemical composition of RAM and bacterial and fungal communities, were clarified in this study. The abundance of rhizosphere microorganisms in different habitats was different, and the content of effective components in plants from different habitats was also significantly different. Rhizosphere microorganisms were found to play an important role in the accumulation of these compounds by analyzing the accumulation of rhizosphere microorganisms and atractylone, polysaccharides, and lactone components. The content of effective components in wild plants was higher than that in artificially cultivated plants, and the abundance of bacteria and fungi that are positively correlated with the content of effective components was also higher than that in artificially cultivated plants. This might be a key reason for the higher accumulation of chemical constituents in wild plants. These results might have potential application value for the rhizosphere of RAM. In the long run, this information could help screen beneficial probiotics as biofertilizers to improve the yield and medicinal value and promote the sustainable development of RAM, as well as lay the foundation for future research on endangered material protection.

## Additional Information and Declarations

### Data Availability

The following information was supplied regarding data availability: The data is available at NCBI: PRJNA869875.
